# Non-coding RNAs and chromatin: key epigenetic factors from spermatogenesis to transgenerational inheritance

**DOI:** 10.1186/s40659-021-00364-0

**Published:** 2021-12-20

**Authors:** Carolina Cheuquemán, Rodrigo Maldonado

**Affiliations:** 1 Núcleo de Ciencias Biológicas, Dirección de Núcleos Transversales, Facultad de estudios Interdisciplinarios, Universidad Mayor, Temuco, Chile; 2 Institute of Anatomy, Histology and Pathology, Faculty of Medicine, Universidad Austral de Chile, Valdivia, Chile

**Keywords:** Epigenetic inheritance, Chromatin, Methylation, Epigenome, Embryonic development, Offspring

## Abstract

Cellular fate and gene expression patterns are modulated by different epigenetic factors including non-coding RNAs (ncRNAs) and chromatin organization. Both factors are dynamic throughout male germ cell differentiation on the seminiferous tubule, despite the transcriptional inactivation in the last stages of spermatogenesis. Sperm maturation during the caput-to-cauda transit on the epididymis involves changes in chromatin organization and the soma-to-germ line transference of ncRNAs that are essential to obtain a functional sperm for fertilization and embryo development. Here, the male environment (diseases, drugs, mental stress) is crucial to modulate these epigenetic factors throughout sperm maturation, affecting the corresponding offspring. Paternal transgenerational inheritance has been directly related to sperm epigenetic changes, most of them associated with variations in the ncRNA content and chromatin marks. Our aim is to give an overview about how epigenetics, focused on ncRNAs and chromatin, is pivotal to understand spermatogenesis and sperm maturation, and how the male environment impacts the sperm epigenome modulating the offspring gene expression pattern.

## Introduction

Nuclear processes are highly regulated by macromolecular complexes composed mainly by proteins and nucleic acids. Every nucleus of human cells contains approximately two meters of DNA that is highly packaged in the form of chromatin in a compartment of 10–15 micrometers. The basic unit of chromatin, the nucleosome, allows this incredible level of compaction where a histone protein octamer (2xH2A, 2xH2B, 2xH3, 2xH4) wraps around 146 DNA base pairs [1]. Besides DNA and protein composition, by the mid-1960 s RNA molecules were found as components of chromatin, which already suggested the important role of RNA in gene expression regulation [2].

Later, new experimental techniques allowed us to define RNAs as fundamental chromatin components. Through protein interactions or by directly interacting with DNA, RNA molecules act as modulators of the integrity of nuclear compartments, chromatin accessibility, chromatin remodeler activity, and gene expression [3–7]. Furthermore, detailed analysis of these interactions showed that RNA molecules can destabilize nucleosomes promoting the eviction of histones H2A-H2B, creating accessible regions probably associated with transcriptional activity [8]. From another perspective, nucleosomes stabilize RNA molecules by forming triple helices (ssRNA+dsDNA) on their entry-exit site, which was shown to be associated with transcriptional activation [9]. These results indicate the relevance of the RNA-chromatin association, which can be defined from different angles, but always connected with gene expression regulation.

**Fig. 1:**
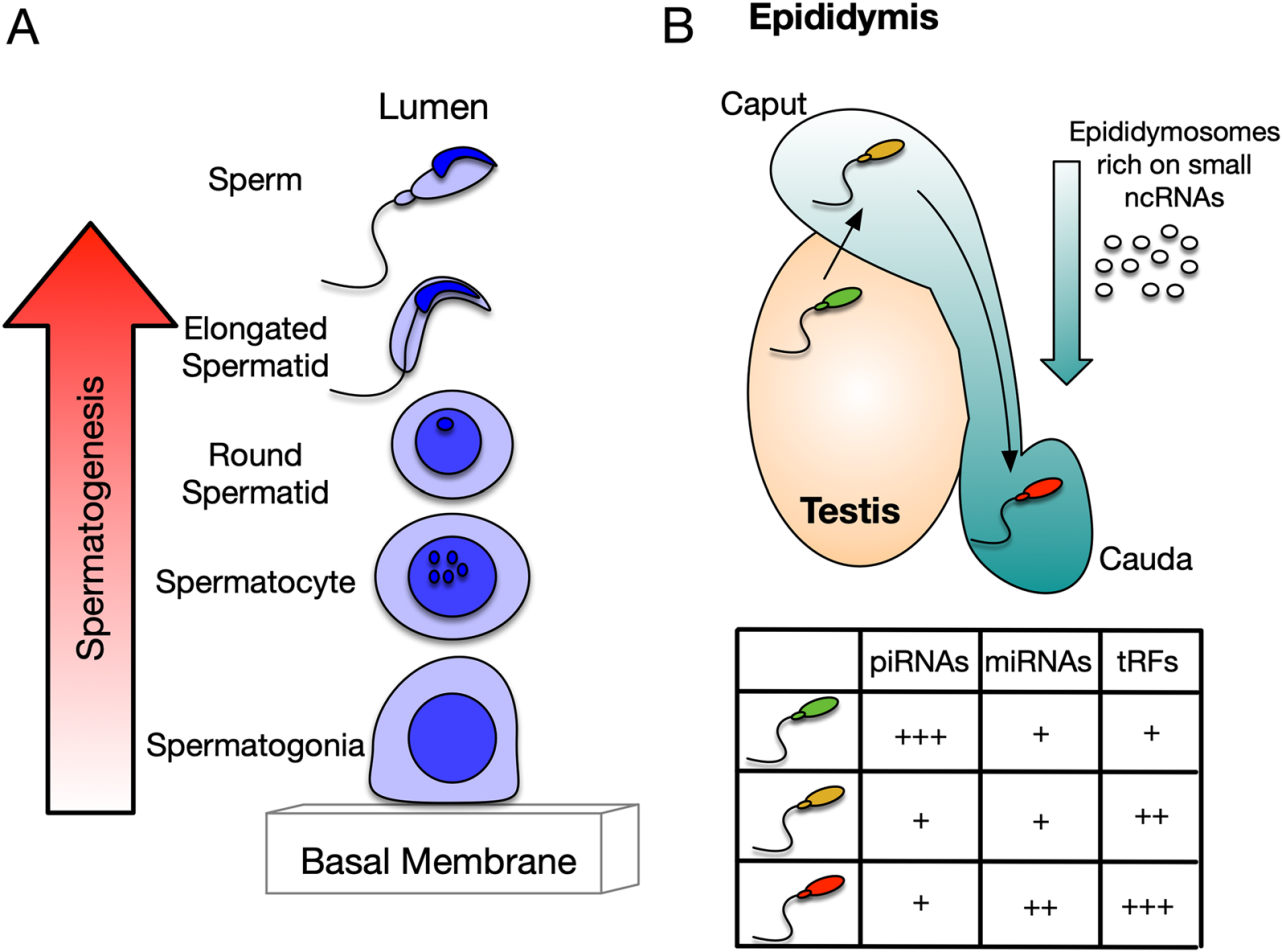
RNA content from germ cells to a mature sperm. **A** Inside the testis, germ cells differentiate from spermatogonia to immature sperm throughout the epithelium of the seminiferous tubules. **B** Immature testicular sperm (green sperm) is released from the tubules reaching the first third of the epididymis, the caput, where are still immature (yellow sperm). Next, sperm travels throughout the epididymis from the caput to the cauda to mature (red sperm). This passage involves a series of input clues from the epididymis, including the transference of small ncRNAs through the epididymosomes modifying the sperm RNA payload shown in the table

Both RNA and chromatin have been fully involved in physiological sperm production followed by their maturation through the male tract. Germ cell differentiation into sperm generates different cellular intermediates (Fig. 1A), which confer some unique features to the testis. One example reflecting this distinctiveness is that the testicular transcriptome is far more complex compared to other organs like the brain or liver [10]. In the testis, germ cell differentiation involves the histone to protamine exchange and transcriptional inactivation during spermiogenesis [11], producing immature sperm cells that are highly enriched on specific non-coding RNAs. Sperm maturation through the male reproductive tract takes place in the epididymis and is completed after several molecular and biochemical changes. One of the most striking features characterizing sperm maturation is the variation in the RNA payload (Fig. 1B). All these physiological modifications are necessary to obtain highly functional spermatozoa for successful embryo developmental competence.

Due to advances in the comprehension of these processes, paternal reproductive influence on offspring has gained attention in recent years. Studies focused on paternal transgenerational inheritance have shown the relevance of epigenetic changes on the paternal phenotype transference. This is mainly driven by the fact that the sperm epigenome escapes reprogramming after fertilization [12]. Thus, environmental influences on the sperm epigenome impact the offspring in various alterations related to paternal nutrition, cancer, mental stress and cognitive disfunction. So far, all these modifications are mostly focused on differential DNA methylated regions, histone retention and enrichment/depletion of ncRNAs. Additionally, the sperm epigenome has also been related with infertility and assisted reproductive techniques (ARTs). Epigenetic factors have been found to be accurate markers and useful biological tools to assess male infertility for pre-conception advice, sperm selection, and during in vitro fertilization. On the other hand, the possible consequences of gamete handling on the epigenome of in vitro derived embryos are discussed.

In this review we want to give an overview of RNA and chromatin features and changes during spermatogenesis, and sperm passage throughout the epididymis for maturation, with a final focus on environmentally induced epigenetic transgenerational inheritance. Understanding that RNA molecules and chromatin, or both associated, are essential components that define specific genomic organizations in sperm, is key to explain their role in controlling gene expression and establishing specific phenotypes in the offspring.

### Epigenetic and transcriptional changes during spermatogenesis

#### Chromatin organization and transcriptional levels

The seminiferous tubule is organized as a complex stratified epithelium where germ cells differentiate into spermatozoa in a process called spermatogenesis. For a more detailed explanation of this process please refer to [13]. During germ cell development, their genetic material is subjected to several changes including replacing the proteins compacting the DNA. This spermatogenic process has been described in three consecutive phases: the first, where mitotic spermatogonia proliferate for self-renewal and give rise to spermatocytes; the second, which continues with meiotic spermatocyte division generating haploid spermatids; and the third called spermiogenesis, where several cytological and biochemical changes transform spermatids into sperm [14].

Throughout the spermatogenic process, germ cell genetic material is packed by canonical and testis-specific histones until the transition from round to elongated spermatids [15]. At this stage, histone tail hyperacetylation relaxes the histone-DNA interactions allowing their exchange by transition proteins during nuclear condensation [16]. Finally, transition proteins are replaced by highly-positive proteins, called protamines, which form tight toroidal complexes that pack around 85–90% of chromatin in secondary spermatids and will remain until sperm formation [15, 17].

All these changes highlight some exceptional features of the testis, given specifically by the germ cells developing inside the seminiferous tubule. The histone epigenetic profiling of genes in mammalian germ cells showed that core poised genes (genes carrying bivalent active H3K4me3 and inactive H3K27me3 marks) are transcriptionally dominant, controlling somatic gene expression patterns and embryonic development of diverse species spanning 300 million years of evolution [18]. This suggests an evolutionary association between germ cell chromatin epigenetics, somatic gene expression modulation, and the embryo gene expression program. Another example for the special features observed in testes arises from a large RNA-seq study comparing different tissues and revealing a higher transcriptional complexity for the testis compared to heart, brain, and liver, in mammals and chicken [10]. This higher complexity level seems to be facilitated by open chromatin states at specific stages, observed as strong H3K4me2 marks on the transcriptional start sites (TSS) of meiotic spermatocytes and post-meiotic round spermatids, which explain the widespread transcriptome of coding and non-coding genes at these cell differentiation stages [10, 19]. Likewise, an assay for transposable-accessible chromatin with sequencing (ATAC-seq) and RNA-seq data showed a dynamic chromatin reorganization and transcriptome diversity, respectively, following the germ cell differentiation of accessible intergenic and intronic regions during the mitosis-to-meiosis transition. Here, de novo formation of accessible regions occurs in autosomes at the stage of pachytene spermatocytes, as well as on sex chromosomes during meiotic sex chromosome inactivation (MSCI). Later when round spermatids are generated, chromatin turns largely inaccessible without the occurrence of de novo accessible regions. Interestingly, at these advanced stages, genes that are not expressed show ATAC signals (namely accessible regions) corresponding with TSS [20]. These results suggest that already at the final stages of spermatogenesis, the chromatin organization is being prepared, or primed, for the sperm role during embryonic development as proposed earlier [21]. In the same line, chromosome conformation capture (3C) data from primate and mice germ cells revealed that chromatin is reorganized during the mitosis-to-meiosis step. Topologically associating domains (TADs), defined primarily in fibroblasts, were dissolved in pachytene spermatocytes and reestablished in round spermatids. Interestingly, a strong effect is mediated by the synaptonemal complex during meiosis on pachytene spermatocytes, which shows a unique chromatin configuration that is tightly associated with transcriptional-correlated compartments [22]. This evidence has been supported by the reorganization of super-enhancers, most probably reflecting changes in chromatin configuration, converging to the dynamism of the transcriptome during mitosis-to-meiosis transition in developing germ cells [20].

The diversity of gene expression levels during spermatogenesis has been examined by single cell RNA-seq combined with bulk RNA-seq, showing a less complex transcriptome in the earlier steps of meiosis and the highest number of expressed genes at the prophase I level in diplotene spermatocytes. Thus, during early spermiogenesis, canonical histones are atypically expressed during round spermatid differentiation, while testis-specific histones and protamines are enriched in late spermatids followed by the lowest number of expressed genes [23]. The morphological changes observed in elongating spermatids therefore occur in consonance with histones’ post-translational modifications and the subsequent chromatin condensation leading to a decrease in the transcriptional activity [23–25]. This latter effect was already described in the mid-1970 s via electron microscopes. Ribonuclear particle complexes of chromatin, RNA, and proteins were observed as transcriptionally active centers (beaded chromatin) in less differentiated cells, compared to the more differentiated ones like late spermatids, where chromatin was described as a “smooth” type restricting the transcription [26]. To overcome the last steps of spermiogenesis under low transcriptional levels, coding and non-coding transcripts are stored in specialized cytoplasmic granules called the chromatoid body, while genes coding for nuclear proteins that are essential for chromatin condensation and sperm motility are still being transcribed [23, 27]. Therefore, the germ cell differentiation that takes place on the seminiferous epithelium from spermatogonia to sperm represents a highly complex process that not only involves large morphological changes, but also an enormous degree of histone modifications, chromatin reorganization and transcriptional dynamics in order to maintain genetic material integrity during the sperm production process.

### Non-coding RNAs on spermatogenic cells

Nuclear processes that modulate the chromatin compaction, transcriptional levels, and functional ncRNAs expression, are highly regulated at different stages of spermatogenesis. One class of ncRNAs described on spermatogenic cells are the endogenous small interfering RNAs (endo-siRNAs), which are produced from naturally-occurring double-strand RNA (dsRNA). The endo-siRNAs are one type of small non-coding RNAs (ncRNAs) involved in the RNA-interference (RNAi) mechanism to modulate gene expression at the post-transcriptional level [28]. endo-siRNAs enrichment has been described on mouse testicular germ cells, with mRNAs as their main predicted targets, but intriguingly, they have one order of magnitude more targets at the genomic DNA level compared to the RNA transcripts [29]. This high degree of genomic complementarity suggests an epigenetic role for endo-siRNAs, as has been reported in other species [30].

Another class of small ncRNAs involved in RNAi, but derived from single stranded RNA, which are abundantly expressed in the germ lines, are the so called Piwi-interacting RNAs (piRNAs) [31]. piRNAs are known to protect germ cell genome integrity by the post-transcriptional gene silencing of transposable element-derived transcripts (TE’s) [32]. In addition to this direct effect over the TE’s, piRNAs have been involved in the de novo methylation of transposable elements and the maintenance of the repressive H3K9me3 mark in long interspersed nuclear elements, both via the recruitment of methyl transferases and other protein factors in male germ cells [33–37]. Therefore, it is not surprising to find piRNAs in the nucleus of murine pachytene spermatocytes, examined by Northern blot and Fluorescence In situ Hybridization [38]. Throughout testicular development and mouse spermatogenesis, piRNAs showed two stage-specific expression peaks. The first peak is described in pro-spermatogonia around birth, finding fetal and postnatal piRNAs. During this period, cells are reprogrammed to maintain them epigenetically uniform and generate their own parental imprinting by de novo methylation. The second peak is observed in pachytene spermatocytes. Here, the interaction between piRNAs piwi proteins is essential for spermatogenesis [39].

Additionally, another class of small ncRNAs, the microRNAs (miRNAs) has been detected in spermatocytes nuclei [38]. miRNAs were the first class of the small ncRNAs discovered and they derive from a stem loop precursor (dsRNA) that is processed by two endonucleases (RNAse III enzymes), first in the nucleus and then in the cytoplasm generating a ∼22 nucleotides molecule [40]. miRNAs are then loaded to the RNA-induced silencing complex (RISC) where they interact with different proteins of the Argonaute (Ago1-4) family, with only the “slicer” endonuclease activity of Ago2 responsible for the mRNA cleavage [41]. Compared to other species, humans have evolutionary gained miRNA families through their duplication from the X chromosome, in combination with their escape from the transcriptional silencing triggered by the MSCI in spermatocytes and spermatids [42, 43]. Using knock-down mice for specific miRNAs it was found that the absence of the miR-106b~25 cluster perturbs normal germ cell development. Although these mice were still fertile, the transcriptome of the early spermatogenic cells from spermatogonia to leptotene/zygotene spermatocytes was deregulated and perceived as a decrease in testis mass and epidydimal sperm content [44]. Using the same approach, the knock-down of the miR-34/449 family generated infertile mice with abnormal spermatogenesis, along with scarce presence of elongating spermatids and spermatozoa on the seminiferous tubule [45]. miRNA profiling on the cells constituting the seminiferous epithelium have revealed testis-specific and germ cells stage-specific miRNAs expression in mammals [46–48]. In humans, stage-specific variations in the germ cells miRNA repertoire have been observed by comparing patients with normal spermatogenesis (azoospermic, OA) against patients with altered spermatogenesis (non-obstructive azoospermia, NOA). Interestingly, in the three germ cells analyzed (spermatogonia, pachytene spermatocytes, and round spermatids), NOA patients with altered spermatogenesis showed an enormous change in small RNA content compared to OA patients. Small non-coding RNA distribution exhibited a dramatic increase of miRNAs, concomitant with the decrease of piRNAs percentage on NOA patients. These RNA biotype composition changes were also accompanied with changes in the expression (up- and down-regulation) of different miRNAs, which in turn generated changes in the expression of essential genes for spermatogenesis in NOA patients [49].

In addition, with the functional expression of miRNAs in germ cells, the Argonaute proteins and the miRNA maturation machinery are highly expressed in testis and male germ cells, specifically the endonuclease Drosha and Ago4 [50]. Ago4 and Ago3 proteins, along with X chromosome-derived miRNAs, were localized to the sex body during prophase I of pachytene spermatocytes in wild type mice. Reduced testis weight and sperm production were observed in mice knock-down for Ago4, most probably explained by the increase in TUNEL-positive cells in the seminiferous epithelium. Moreover, increased Ago3 expression was observed when Ago4 was not expressed, suggesting a possible compensation mechanism between them. Ago4 knock-down mice showed alterations in the formation of the sex body, the localization of key proteins that compose these structures, as well as in the deposition of silencing marks on sexual chromosomes, affecting the MSCI process in pachytene spermatocytes. The loss of Ago4 also resulted in the downregulation of several miRNAs and spermatogonia prematurely entering meiosis. All these results suggest an epigenetic role of Ago proteins and miRNAs in the transcriptional silencing process during spermatogenesis [51]. A defined role for nuclear miRNAs and Ago proteins on spermatogenic cells is still unexplored. Nevertheless, their influence in the deposition of chromatin marks on male germ cells is supported by the role of miRNAs and Ago4 as the effector protein for the RNA-dependent DNA methylation process in other human cells [52–54].

Hence, the spermatogenic process ends up with an immature sperm cell that is transcriptionally inactive, but with several chromatin accessible regions corresponding with TSS and loaded with a considerable number of small RNA molecules (Fig. 1B).

### Fine tuning the sperm epigenome during epididymal transit

Non-functional spermatozoa released from testes follow an essential caput-to-cauda transit through the epididymis, which takes 2–6 days in humans [55] and 10–13 days in rodents, in order to mature, gain motility, and the capacity to fertilize an oocyte [56–58]. During this passage, sperm is subjected to different “epididymal-environmental clues” that aid in their maturation including RNAses, DNAses, proteases, glycan-modifying enzymes, changes in membrane lipid composition and protein phosphorylation [59–62]. This transit also involves the epididymis-to-sperm transference of various molecules through vesicles that fuse to the sperm, called epididymosomes, which include signaling molecules, proteins, and different small RNAs [63–65]. This phenomenon modifies the spermatozoa small RNA content, described in the field as the “RNA payload”. Comparing the small RNA payload of mature sperm from the cauda epididymis versus immature testicular spermatozoa, it was found a differential RNA-type enrichment depending on the sperm maturity levels. Mature cauda sperm was highly enriched in 29–34 nt RNAs, while the testicular sperm in 26–32 nt RNAs that correspond mainly to piRNAs [64, 66]. These 29–34 nt RNAs found in mature spermatozoa, were identical in their 5` ends, differing only on their 3` sequences and aligned to different transference RNA (tRNA) loci. Thus, by analyzing the sequences against tRNA databases, all the small RNAs matched to the 5` halves of specific tRNAs with defined cleavage sites on the anticodon loop [66]. Interestingly, all those tRNA-derived fragments (tRFs) found in mature spermatozoa, but not in testicular sperm, showed a high correlation with the small RNA content of epididymosomes, suggesting that epididymosomes were the source for this variation on the sperm RNA payload [64] (Fig. 1B). Further studies corroborated tRF transference from the epididymal epithelial cells to spermatozoa through the epididymosomes, including the in vitro transferring of small RNAs to sperm using thiol(S)-linked alkylation for the metabolic sequencing of RNA (SLAM-seq) on a genetically modified mouse that allows the tissue-specific 4-thiouracil RNA-labeling [67–69].

Similarly, the RNA payload is also fine-tuned by the differential transference of miRNAs throughout the epididymis [70]. The sequencing of small RNAs focusing the read alignment on miRNAs found that sperm obtained from different portions of the epididymis showed different miRNA signatures, where around 100 miRNAs are lost, and the same number are acquired during the caput to cauda transit (Fig. 1B). Moreover, preliminary analysis of the miRNA sequencing data supported by the presence of the processing machinery suggested that a subset of miRNAs mature from the corresponding precursors during epididymal transit [70].

Sperm RNA content variations during caput-to-cauda transit have been functionally analyzed by sperm capacity for subsequent embryo development. Caput-derived sperm embryos showed alterations during preimplantation, most probably due to the overexpression of key regulatory (RNA- and chromatin-associated) factors. Strikingly, this preimplantation failure was reverted by microinjecting miRNAs, but nor tRFs, purified from cauda epididymosomes along with caput-derived sperm during the Intracytoplasmic Sperm Injection (ICSI) procedure [71]. These findings therefore disclose some of the features that modulate the variations of the small RNA payload following maturation through the epididymis, which can be directly associated to fertilization, embryo development and appropriate implantation.

In addition to RNA payload changes, the passage throughout the epididymis also modifies sperm chromatin architecture and accessibility. During this phase, intra- and inter-protamine disulphide bonds are promoted, increasing chromatin compaction levels to maintain and protect genetic material integrity [72, 73]. This general chromatin condensation process seems to be affected in mice knocked-down for the cannabinoid receptor CB1, where alterations in histone displacement and protamine disulphide bonds were described [74, 75]. The mechanisms associated to CB1 receptor loss and altered chromatin condensation are associated to a decrease in intratesticular estrogen levels and histone H4 acetylation [74]. Nevertheless, the factors and signals at the molecular level to understand the role of CB1 during the chromatin condensation throughout the epididymal maturation of the sperm need to be further investigated.

Sperm chromatin fragmentation is a natural phenomenon occurring throughout sperm transit in the epididymis tract. During this process, defective sperm are eliminated in an apoptotic-like process strongly influenced by the luminal epididymal (and *vas deferens*) fluids to degrade DNA [76–79].

Mature cauda sperm chromatin has been described to retain 2-15% histones compared to somatic cells, depending on the technical approach employed for determination [21, 24, 80–82]. Histone methylation mark occupancy defined by chromatin immunoprecipitation sequencing (ChIP-Seq), revealed that most of the H3K4me2/3 marks are equally localized between sperm and somatic cells. As the exception, a considerable number of H3K4me2/3 sperm-specific sites were found that mostly reflect the transcriptional activity in the final stages of spermatogenesis [81]. At the chromatin accessibility level, MNase-seq of mice sperm compared to embryonic stem cells showed nucleosomes occupancy over large gene-poor regions, with a small subset localized over promoter for developmental regulators [80]. A similar methodology that sequenced only the mono-nucleosomal fraction found that sperm nucleosomes are located mainly on centromere repeats and retrotransposons, with a particular depletion in regulatory elements like 5´- and 3´-untranslated regions (UTRs), TSS, and transcriptional termination sites (TTS) [82]. Additionally, MNase footprinting showed the first evidence for CTCF-chromatin interaction, suggesting a role in sperm-chromatin architectural organization [80]. Further studies in mice, performing ATAC-seq combined with ChIP-seq, showed that nucleosomes are precisely positioned flanking promoters containing active marks, specifically the 60% of sperm promoters containing H3K4me3, H3K27Ac, H3K9Ac [21]. In addition, when integrated with high-throughput chromosome conformation capture (HiCi) data, it has been found that CTCF and Cohesin organize the architecture of topologically associated domain (TADs) of the mice sperm genome [21, 83]. In humans, a different organization has been described for chromatin in sperm. Here, chromatin lack TADs and CTCF, and only after fertilization occurs, TADs start to be established and CTCF expressed, both specifically at the zygotic genome activation (ZGA) stage [84]. A subset of the sperm active promoters corresponds with genes actively transcribed on round spermatids, while the remaining active promoters, in addition to enhancers and super-enhancers, mirror the transcriptional activity of genes found in mouse embryonic stem cells (mESCs) and mouse embryonic fibroblasts (MEFs). This suggest that epididymis-cauda sperm harbor an intricate epigenome, which reflects the transcriptional state of immature states and is also primed for later stages during embryonic development modeling future expression patterns [21].

Sperm heads are composed mainly of a tiny cytoplasmic fraction, the acrosomal vesicle on the apical section, and the nucleus on the central region as the largest head compartment. This indicates that to a certain degree, chromatin and RNA molecules could be directly or indirectly associated in the sperm nucleus, as has been described extensively in somatic cells by different methods [85]. Non-radioactive in-situ hybridization found U1 and U2 small nuclear RNAs on epididymal sperm nuclei [86]. Sperm RNA-seq and a further classification of the transcripts found a subset of RNAs that were primarily defined as chromatin-associated (caRNA) in humans [87, 88]. A differential RNA purification during aqueous-organic phase separation with commonly used phenol-guanidine isothiocyanate solution mixed with chloroform followed by sequencing identified two categories of RNA in mature cauda sperm. One category corresponds to the “free” RNA fraction purified from the aqueous phase, while the second category was obtained from the interphase after protein and DNA digestion, described as DNA-associated RNA. RNA sequencing showed that both fractions contain different subsets of coding and non-coding transcripts, where the main fraction of DNA-associated RNAs was sensitive to RNAseH, suggesting the presence of ssRNA-ssDNA hybrids (R -Loops) that probably correspond to nascent transcripts “frozen” during transcriptional activity throughout spermatogenesis [89]. These results provide a potential way to understand how both chromatin and RNA coexist and associate in the compacted sperm nucleus. Nevertheless, more studies are needed to clearly define the nature and molecular determinants for DNA-associated transcripts in mature sperm.

### Molecular mechanisms for sperm epigenetic transgenerational inheritance

Epigenetics refers to changes in molecular factors and processes around the DNA that regulate genome activity independent of DNA sequence that are mitotically stable [90]. These changes include DNA methylation, histone modifications, non-coding RNAs, RNA methylation, and chromatin structure, which all together make up the epigenome [91]. Epigenetic transgenerational inheritance corresponds to the germline-mediated inheritance of epigenetic information between generations in the absence of continued direct environmental influences that leads to phenotypic variation [92, 93]. While there is a coordinated reprogramming between paternal and maternal genomes upon fertilization [94], sperm epigenetic changes escape the reprogramming process and are involved in the transmission of transgenerational phenotypes [12]. Therefore, paternal environmental factors before conception and during spermatogenesis can determine the health of the offspring in later life.

Nowadays, three main molecular mechanisms for environmentally induced sperm epigenetic variations are described in scientific literature: (1) DNA methylated regions (DMRs), (2) Differential histone retention sites (DHRs), and (3) ncRNAs.

DNA methylation at cytosines adjacent to guanine (CpG) sites was the first established epigenetic mark. High density CpG islands and low density CpG regions, termed CpG deserts have important roles in genome activity regulation [95]. Sperm DMRs have been functionally associated to signaling, transcription, metabolism and receptors [12, 96, 97]. Differential DMRs are first induced during the fetal gonadal sex determination period [98]. DMRs later arise throughout the development of pro-spermatogonia, spermatogonia and pachytene spermatocytes [12].

In the same line, DHRs position in the genome correlates with genes associated with cell signaling, metabolism and transcription, which may alter zygote and embryo development [90, 91, 99]. Rats exposed to agricultural pesticides showed that DMRs have a positive correlation with DHRs and ncRNAs expression [100], suggesting both histone retention site guiding by DNA methylation and RNA-directed DNA methylation [101]. In addition, pericentric histone retention was found to be directed by nuclear RNAs in mature spermatozoa, specifically through the RNA-binding motif of the testis-specific histone variant H2A.L.2 [102].

On the other side, different long non-coding RNAs (lncRNAs) have been found to be enriched in sperm compared to round spermatids. Interestingly, those lncRNAs seem to target transcripts of genes related to nucleic acid metabolism, protein modification, chromatin and histone modification, heterocycle compound metabolic, sperm function and spermatogenesis [103]. Similarly, a transgenic mouse model overexpressing the histone demethylase KDM1A showed that H3K4me3, but not H3K27me3, was almost completely retained after fertilization, and therefore that sperm H3K4me3 marks can be transmitted transgenerationally [104]. All this evidence lead us to think about a complex and concerted interaction between chromatin and ncRNAs in mature sperm, in order to control the embryonic gene expression program and the establishment of specific phenotypes in progeny.

An environmental factor that modifies offspring epigenomes, especially related to obesity, is paternal diet [105–107]. Using a high fat diet (HFD)-induced obesity mouse model, altered histone H3 occupancy was found in regulatory genes implicated in embryo developmental processes and differential H3K4me1 marks compared to control mice [108]. In humans, obese men present an altered sperm tsRNA content [109], while the up-regulation of sperm mir-19b is associated to men that ate a Western-style diet [110]. RNA-seq analyses of early embryos after the injection of sperm tsRNAs from a high fat diet-fed father revealed a decrease in the expression of metabolic regulation-related genes in both early embryos and the pancreatic islets of offspring [111]. In the same way, sub-optimal paternal nutrition (low protein diet) has a strong impact on offspring well-being by programming cardiovascular function over successive generations [112].

Another inheritable effect driven by epigenetic changes in sperm was shown in mice knockout for the histone demethylase *Kdm6a*. Here, the deletion of this tumor suppressor increased the incidence of tumors in their wild type offspring. Knockout mice sperm showed higher levels of methylation in both H3K27 and DNA at specific loci. Interestingly, some of these DNA hypermethylated regions in sperm were also observed in somatic cells of the wild type offspring, probably perturbing gene expression programs that resulted in elevated tumor incidence in the progeny [113]. Another study analyzing the relevance of sperm-derived transcripts during embryo development showed that sperm-specific transcripts are highly expressed during ZGA [114]. Sperm-specific lncRNAs enriched with H3K4me3 in their promoters were also found deregulated in different cancers, exhibiting a direct correlation between their H3K4me3 promoter enrichment and increased expression in cancer. These results suggest the oncogenic properties of the sperm-specific lncRNAs and its potential use as diagnostic and prognostic markers for several cancers [114]. As epigenetic modifications are reversible, many drugs targeting epigenetic modifying proteins are frequently used in cancer treatment for males at reproductive age; however, its effects in germline epigenome and subsequent child health are limited [115].

Transgenerational inheritance has also been related to mental disease and cognitive function in offspring brains. Parental stress exposure influences the risk of stress reactivity and post-traumatic stress disorder (PTSD) risk in subsequent generations, contributing to the developmental programming of the hypothalamic-pituitary-adrenal (HPA) stress axis [116]. A mouse model of restrain stress showed increased methylation levels in the promoter of *Sfmbt2* gene in sperm, which led to the downregulation of miR466b-3p in the liver cells of the offspring. This miRNA targets the 3´UTR of the gluconeogenic enzyme phosphoenolpyruvate carboxykinase (PEPCK) mRNA. Therefore, the decreased expression of this miRNA increases PEPCK levels and induces hyperglycemia [117]. Consequently, an in silico analysis creating a model for transgenerational inheritance reinforced positive feedback for DNA methylation in the sperm *Sfmbt2* promoter as a possible mechanism to mediate parental psychological stress reprogramming in offspring [118]. Similarly, altered miRNAs content in response to enviromental stress in early life is transmitted through sperm to their offspring. Moreover, injection of sperm RNAs from traumatized males into fertilized wild-type oocytes reproduced the behaviour and metabolic stress response phenotype in the resulting offspring [119]. In contrast, environmental enrichment leads to intergenerational inheritance of high cognitive abilities by sperm miRNAs, especially miRs 212/132, which enhances synaptic plasticity and cognition in the next generation and supports the crucial role of physical exercise and cognitive training for preventing mental disorders [120]. Another inherited cognitive defect associated with epigenetic changes is related to the increased risk of autism spectrum disorder (ASD) with paternal aging [121, 122]. Using a mouse model of paternal aging characterized by defective communication, it was discovered that sperm from aged mice were hypomethylated compared to young mice, specifically in genes targeted by RE1-silencing transcription factor (REST) and the neuron-restrictive silencer factor (NRSF). Consequently, the expression analysis in the offspring showed an enrichment of those sperm hypomethylated REST/NRSF-targeted genes in developing brain cells. Offspring from aged fathers also presented precocious neurogenesis and reduced cortical thickness of the primary motor cortex, explaining the altered communication [122].

Sperm epigenetic variations are directly associated to embryo development and ARTs. Sperm-borne miRNAs and endo-siRNAs are important to control transcriptomic homeostasis in fertilized oocytes, zygotes, and two-cell embryos. sncRNA-deficient sperm displayed a significant reduction in embryo developmental potential, which could be rescued by injecting sperm-derived total or small RNAs into embryos during intracytoplasmic sperm injection (ICSI) [71, 123]. Accordingly, sperm RNAs enrichment might represent a novel way to improve fertility rates during gamete handling, in vitro fertilization (IVF) or ICSI procedures.

By contrast, ARTs can induce epigenetic variation that might be transmitted to the next generation. Specific and functional epigenetic changes in DNA methylation and H3K4me3 patterns from in vitro derived embryos demonstrate that the ICSI technique might interfere with processes associated with skeletal and immune systems in offspring [124]. This could be related to the fact that during ICSI sperm is artificially introduced into the oocyte avoiding natural sperm selection, and it is therefore not the “best biological” sperm in terms of function and quality which finally penetrates the oocyte. This reinforces the need for further research into sperm epigenetics and transgenerational inheritance on subsequent in vitro derived embryos after ARTs.

In recent years, conventional sperm quality analyses have tried to integrate new molecular biology techniques to increase human fertility. However, these new assays are not used routinely. Clinical sperm evaluations found that the epigenetic mark H3K4me2 is negatively correlated with sperm concentration, motility and mitochondrial activity in humans, and has therefore been considered a marker for sperm quality assessment [125]. Similarly, phosphorylated serine 1 of H4 (HS1ph) has been suggested as a epigenetic marker for sperm maturity during spermatogenesis. HS1ph levels are significantly decreased in healthy mature sperm nuclei, and high levels of this mark were closely associated with sperm immaturity and infertility [126]. Regarding early embryo quality, a specific sperm miRNA profile was described as a potential marker to screen high-quality sperm in order to improve IVF success rates. Sperm samples with high hsa-mir-191 expression had a higher fertilization rate, blastocyst rate and high-quality embryo rate [127]. Patients with normal semen analyses but not capable of inducing a pregnancy have shown under-expressed DNA repair genes (*APLF, CYB5R4, ERCC4* and *TNRFSF21*) and apoptosis-modulating genes (*MORC1, PIWIL1* and *ZFAND6*), with an inverse correlation with age and DNA fragmentation [128]. In these patients, 16 lncRNA genes were also completely downregulated, with most appearing to guide chemical modification of other RNAs, influence methylation, and modulate messenger RNA stability and translation [128]. In a similar biomedical model, boars with high quality semen parameters but lower fertility success have shown that most of the DMRs were hypermethylated on genes related to spermatogenesis, sperm function, fertilization and fertility/prolificacy. Additionally, they showed the same disregulation of sperm miR-153 that occurs in humans, affecting fertilization and embryo rates after IVF [129].

**Fig. 2:**
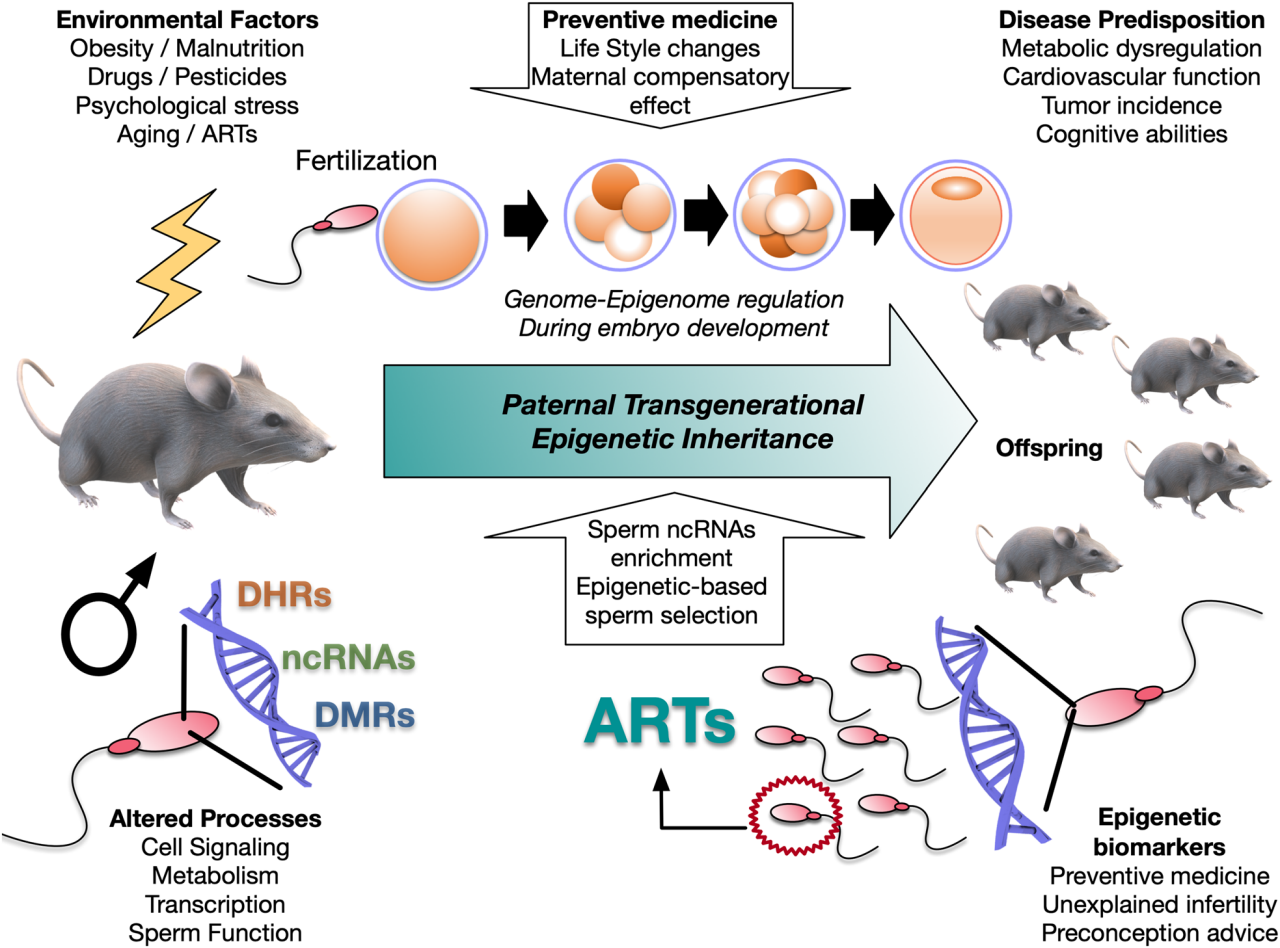
Environmental factors determining transgenerational epigenetic inheritance affecting offspring health. Sperm epigenome alterations due to environmental cues are transferred to the offspring. These inherited epigenetic signals can be useful biomarkers for disease diagnosis and treatment in preventive medicine for offspring. Paternal transgenerational epigenetic inheritance can be compensated by lifestyle and maternal nurture. Epigenetic analyses for sperm selection and in vitro culture enrichment with ncRNAs can be useful to improve fertility rates and embryo quality during ARTs

Altogether, these data demonstrate that sperm epigenetics is relevant for fertility, embryo development and transgenerational inheritance (Fig. 2). Understanding the epigenetics in reproduction will contribute to design rational therapeutic options [130], to accurately assess fertility status [131, 132], predict the impact of parental experiences [133] and lifestyle on epigenetic paternal transgenerational inheritance [134].

## Conclusions

This literature review highlights the importance of regulatory ncRNAs, histone modifications and chromatin accessibility during spermatogenesis to obtain functional sperm and its implications on epigenetic paternal transgenerational inheritance.

## Data Availability

Not applicable.

## Abbreviations

ARTs: Assisted reproductive techniques
ICSI: Intracytoplasmic sperm injection
TSS: Transcriptional start sites
MSCI: Meiotic sex chromosome inactivation
TADs: Topologically associating domains
piRNAs: Piwi-interacting RNAs
DMRs: DNA methylated regions
DHRs: Differential histone retention sites
ASD: Autism spectrum disorder
PTSD: Post-traumatic stress disorder
IVF: In vitro fertilization

## References

[1] Luger K, Mäder AW, Richmond RK, Sargent DF, Richmond TJ (1997). Crystal structure of the nucleosome core particle at 2.8 Å resolution. Nature.

[2] Huang RC, Bonner J (1965). Histone-bound RNA, a component of native nucleohistone. Proc Natl Acad Sci U S A.

[3] Rodríguez-Campos A, Azorín F (2007). RNA is an integral component of chromatin that contributes to its structural organization. PLoS One.

[4] Schubert T, Pusch MC, Diermeier S, Benes V, Kremmer E, Imhof A (2012). Df31 protein and snoRNAs maintain accessible higher-order structures of chromatin. Mol Cell.

[5] Caudron-Herger M, Rippe K (2012). Nuclear architecture by RNA. Curr Opin Genet Dev.

[6] Li X, Fu XD (2019). Chromatin-associated RNAs as facilitators of functional genomic interactions. Nat Rev Genet.

[7] Saldaña-Meyer R, Rodriguez-Hernaez J, Escobar T, Nishana M, Jácome-López K, Nora EP (2019). RNA interactions are essential for CTCF-mediated genome organization. Mol Cell.

[8] Fujita R, Yamamoto T, Arimura Y, Fujiwara S, Tachiwana H, Ichikawa Y (2020). Nucleosome destabilization by nuclear non-coding RNAs. Commun Biol.

[9] Maldonado R, Schwartz U, Silberhorn E, Längst G (2019). Nucleosomes stabilize ssRNA-dsDNA triple helices in human cells. Mol Cell.

[10] Soumillon M, Necsulea A, Weier M, Brawand D, Zhang X, Gu H (2013). Cellular source and mechanisms of high transcriptome complexity in the mammalian testis. Cell Rep.

[11] Rathke C, Baarends WM, Awe S, Renkawitz-Pohl R (2014). Chromatin dynamics during spermiogenesis. Biochim Biophys Acta Gene Regul Mech.

[12] Skinner MK, Nilsson E, Sadler-Riggleman I, Beck D, Ben Maamar M, McCarrey JR (2019). Transgenerational sperm DNA methylation epimutation developmental origins following ancestral vinclozolin exposure. Epigenetics.

[13] Griswold MD (2016). Spermatogenesis: the commitment to Meiosis. Physiol Rev.

[14] Clermont Y (1972). Kinetics of spermatogenesis in mammals: seminiferous epithelium cycle and spermatogonial renewal. Physiol Rev.

[15] Bao J, Bedford MT (2016). Epigenetic regulation of the histone-to-protamine transition during spermiogenesis. Reproduction.

[16] Rousseaux S, Faure A, Khochbin S (2005). Establishment of male-specific epigenetic information. Gene.

[17] Meistrich ML, Mohapatra B, Shirley CR, Zhao M (2003). Roles of transition nuclear proteins in spermiogenesis. Chromosoma.

[18] Lesch BJ, Silber SJ, Mccarrey JR, Page DC (2016). Parallel evolution of male germline epigenetic poising and somatic development in animals. Nat Genet.

[19] Kimmins S, Sassone-Corsi P (2005). Chromatin remodelling and epigenetic features of germ cells. Nature.

[20] Maezawa S, Yukawa M, Alavattam KG, Barski A, Namekawa H (2018). Dynamic reorganization of open chromatin underlies diverse transcriptomes during spermatogenesis. Nucleic Acids Res.

[21] Jung YH, Sauria MEG, Lyu X, Cheema MS, Ausio J, Taylor J (2017). Chromatin states in mouse sperm correlate with embryonic and adult regulatory landscapes article chromatin states in mouse sperm correlate with embryonic and adult regulatory landscapes. Cell Rep.

[22] Wang Y, Wang H, Wang Y, Wang H, Zhang Y, Du Z (2019). Reprogramming of meiotic chromatin architecture during spermatogenesis article reprogramming of meiotic chromatin architecture during spermatogenesis. Mol Cell.

[23] Ernst C, Eling N, Martinez-Jimenez CP, Marioni JC, Odom DT (2019). Staged developmental mapping and X chromosome transcriptional dynamics during mouse spermatogenesis. Nat Commun.

[24] Ooi SL, Henikoff S (2007). Germline histone dynamics and epigenetics. Curr Opin Cell Biol.

[25] Tatehana M, Kimura R, Mochizuki K, Inada H, Osumi N (2020). Comprehensive histochemical profiles of histone modification in male germline cells during meiosis and spermiogenesis: comparison of young and aged testes in mice. PLoS One.

[26] Kierszenbaum AL, Tres LL (1975). Structural and transcriptional features of the mouse spermatid genome. J Cell Biol.

[27] Meikar O, Vagin VV, Chalmel F, Sõstar K, Lardenois A, Hammell M (2014). An atlas of chromatoid body components. RNA.

[28] Watanabe T, Totoki Y, Toyoda A, Kaneda M, Kuramochi-Miyagawa S, Obata Y (2008). Endogenous siRNAs from naturally formed dsRNAs regulate transcripts in mouse oocytes. Nature.

[29] Song R, Hennig GW, Wu Q, Jose C, Zheng H, Yan W (2011). Male germ cells express abundant endogenous siRNAs. Proc Natl Acad Sci U S A.

[30] Claycomb JM (2014). Ancient endo-siRNA pathways reveal new tricks. Curr Biol.

[31] Weick EM, Miska EA, piRNAs (2014). From biogenesis to function. Development.

[32] Ernst C, Odom DT, Kutter C (2017). The emergence of piRNAs against transposon invasion to preserve mammalian genome integrity. Nat Commun.

[33] Aravin AA, Sachidanandam R, Bourc’his D, Schaefer C, Pezic D, Toth KF (2008). A piRNA pathway primed by individual transposons is linked to de novo DNA methylation in mice. Mol Cell.

[34] Pezic D, Manakov SA, Sachidanandam R, Aravin AA (2014). piRNA pathway targets active LINE1 elements to establish the repressive H3K9me3 mark in germ cells. Genes Dev.

[35] Watanabe T, Tomizawa S, Mitsuya K, Totoki Y, Yamamoto Y, Kuramochi-Miyagawa S (2011). Role for piRNAs and Noncoding RNA in de Novo DNA methylation of the imprinted. Science.

[36] Schöpp T, Zoch A, Berrens RV, Auchynnikava T, Kabayama Y, Vasiliauskaitė L (2020). TEX15 is an essential executor of MIWI2-directed transposon DNA methylation and silencing. Nat Commun.

[37] Zoch A, Auchynnikava T, Berrens RV, Kabayama Y, Schöpp T, Heep M (2020). SPOCD1 is an essential executor of piRNA-directed de novo DNA methylation. Nature.

[38] Marcon E, Babak T, Chua G, Hughes T, Moens PB (2008). miRNA and piRNA localization in the male mammalian meiotic nucleus. Chromosom Res.

[39] Fu Q, Wang PJ (2014). Mammalian piRNAs. Spermatogenesis.

[40] Bartel DP (2018). Metazoan MicroRNAs. Cell.

[41] Meister G, Landthaler M, Patkaniowska A, Dorsett Y, Teng G, Tuschl T (2004). Human Argonaute2 mediates RNA cleavage targeted by miRNAs and siRNAs. Mol Cell. julio de.

[42] Meunier J, Lemoine F, Soumillon M, Liechti A, Weier M, Guschanski K (2013). Birth and expression evolution of mammalian microRNA genes. Genome Res.

[43] Song R, Ro S, Michaels JD, Park C, Mccarrey JR, Yan W (2009). Many X-linked microRNAs escape meiotic sex chromosome inactivation. Nat Genet.

[44] Hurtado A, Palomino R, Georg I, Lao M, Real FM, Carmona FD (2020). Deficiency of the onco-miRNA cluster, miR-106b~25, causes oligozoospermia and the co- operative action of miR-106b~25 and miR-17~92 is required to maintain male fertility. Hum Reprod.

[45] Song R, Walentek P, Sponer N, Klimke A, Lee JS, Dixon G (2014). MiR-34/449 miRNAs are required for motile ciliogenesis by repressing cp110. Nature.

[46] Wang L, Xu C (2015). Role of microRNAs in mammalian spermatogenesis and testicular germ cell tumors. Reproduction.

[47] Chen X, Che D, Zhang P, Li X, Yuan Q, Liu T (2017). Profiling of miRNAs in porcine germ cells during spermatogenesis. Reproduction.

[48] Liu Y, Niu M, Yao C, Hai Y, Yuan Q, Liu Y (2015). Fractionation of human spermatogenic cells using STA-PUT gravity sedimentation and their miRNA profiling. Sci Rep.

[49] Yao C, Yuan Q, Niu M, Fu H, Zhou F, Zhang W (2017). Distinct expression profiles and novel targets of MicroRNAs in human spermatogonia, pachytene spermatocytes, and round spermatids between OA patients and NOA patients. Mol Ther Nucleic Acids.

[50] González-González E, López-Casas PP, del Mazo J (2008). The expression patterns of genes involved in the RNAi pathways are tissue-dependent and differ in the germ and somatic cells of mouse testis. Biochim Biophys Acta.

[51] Modzelewski AJ, Holmes RJ, Hilz S, Grimson A, Cohen PE (2012). AGO4 regulates entry into meiosis and influences silencing of sex chromosomes in the male mouse germline. Dev Cell.

[52] Morris KV, Chan SW, Jacobsen SE, Looney DJ (2004). Small interfering RNA—induced transcriptional gene silencing in human cells. Science.

[53] Tan Y, Zhang B, Wu T, Skogerbo G, Zhu X, Guo X (2009). Transcriptional inhibition of Hoxd4 expression by noncoding RNAs in human breast cancer cells. BMC Mol Biol.

[54] Chalertpet K, Pin-On P, Aporntewan C, Patchsung M, Ingrungruanglert P, Israsena N (2019). Argonaute 4 as an effector protein in RNA-directed DNA methylation in human cells. Front Genet.

[55] Turner TT (2008). De Graaf’s thread: the human epididymis. J Androl.

[56] Sullivan R, Mieusset R (2016). The human epididymis: its function in sperm maturation. Hum Reprod Update.

[57] Hess RA. Spermatogenesis: an overview. In: Knobil ED. NJ, ed. Encyclopedia of Reproduction. New York, NY: Academic Press; 1999. pp. 539–545.

[58] Cornwall GA (2009). New insights into epididymal biology and function. Hum Reprod Update.

[59] Krutskikh A, Poliandri A, Cabrera-Sharp V, Dacheux JL, Poutanen M, Huhtaniemi I (2012). Epididymal protein Rnase10 is required for posttesticular sperm maturation and male fertility. FASEB J.

[60] Jones R (2004). Sperm survival versus degradation in the mammalian epididymis: a hypothesis. Biol Reprod.

[61] Tulsiani DRP (2006). Glycan-modifying enzymes in luminal fluid of the mammalian epididymis: an overview of their potential role in sperm maturation. Mol Cell Endocrinol.

[62] Gervasi MG, Visconti PE (2017). Molecular changes and signaling events occurring in spermatozoa during epididymal maturation. Andrology.

[63] Nätt D, Öst A (2020). Male reproductive health and intergenerational metabolic responses from a small RNA perspective. J Intern Med.

[64] Sharma U, Conine CC, Shea JM, Boskovic A, Derr AG, Bing XY (2016). Biogenesis and function oftRNA fragments during sperm maturation and fertilization in mammals. Science.

[65] Koch S, Acebron SP, Herbst J, Hatiboglu G, Niehrs C (2015). Post-transcriptional Wnt signaling governs epididymal sperm maturation. Cell.

[66] Peng H, Shi J, Zhang Y, Zhang H, Liao S, Li W (2012). A novel class of tRNA-derived small RNAs extremely enriched in mature mouse sperm. Cell Res.

[67] Gay L, Miller MR, Ventura PB, Devasthali V, Vue Z, Thompson HL (2013). Mouse TU tagging: a chemical/genetic intersectional method for purifying cell type-specific nascent RNA. Genes Dev.

[68] Herzog VA, Reichholf B, Neumann T, Rescheneder P, Bhat P, Burkard TR (2017). Thiol-linked alkylation of RNA to assess expression dynamics. Nat Methods.

[69] Sharma U, Sun F, Conine CC, Reichholf B, Kukreja S, Herzog VA (2018). Small RNAs are trafficked from the epididymis to developing mammalian sperm. Dev Cell.

[70] Nixon B, Stanger SJ, Mihalas BP, Reilly JN, Anderson AL, Holt JE (2015). The MicroRNA signature of mouse spermatozoa is substantially modified during epididymal maturation. Biol Reprod.

[71] Conine CC, Sun F, Song L, Rivera-Pérez JA, Rando OJ (2018). Small RNAs gained during epididymal transit of sperm are essential for embryonic development in mice. Dev Cell.

[72] Saowaros W, Panyim S (1979). The formation of disulfide bonds in human protamines during sperm maturation. Experientia.

[73] Marushige Y, Marushige K (1975). Transformation of sperm during formation and maturation of rat spermatozoa. J Biol Chem.

[74] Chioccarelli T, Manfrevola F, Porreca V, Fasano S, Altucci L, Pierantoni R (2020). The cannabinoid receptor CB1 stabilizes sperm chromatin condensation status during epididymal transit by promoting disulphide bond formation. Int J Mol Sci.

[75] Chioccarelli T, Cacciola G, Altucci L, Lewis SEM, Simon L, Ricci G (2010). Cannabinoid receptor 1 influences chromatin remodeling in mouse spermatids by affecting content of transition protein 2 mRNA and histone displacement. Endocrinology.

[76] Xie P, Keating D, Parrella A, Cheung S, Rosenwaks Z, Goldstein M (2020). Sperm genomic integrity by TUNEL varies throughout the male genital tract. J Urol.

[77] Suganuma R, Yanagimachi R, Meistrich ML (2005). Decline in fertility of mouse sperm with abnormal chromatin during epididymal passage as revealed by ICSI. Hum Reprod.

[78] Gawecka JE, Boaz S, Kasperson K, Nguyen H, Evenson DP, Ward WS (2015). Luminal fluid of epididymis and vas deferens contributes to sperm chromatin fragmentation. Hum Reprod.

[79] Shaman JA, Prisztoka R, Ward WS (2006). Topoisomerase IIB and an extracellular nuclease interact to digest sperm DNA in an apoptotic-like manner. Biol Reprod.

[80] Carone BR, Hung JH, Hainer SJ, Chou M, Te, Carone DM, Weng Z (2014). High-resolution mapping of chromatin packaging in mouse embryonic stem cells and sperm. Dev Cell.

[81] Brykczynska U, Hisano M, Erkek S, Ramos L, Oakeley EJ, Roloff TC (2010). Repressive and active histone methylation mark distinct promoters in human and mouse spermatozoa. Nat Struct Mol Biol.

[82] Samans B, Yang Y, Krebs S, Sarode GV, Blum H, Reichenbach M (2014). Uniformity of nucleosome preservation pattern in mammalian sperm and Its connection to repetitive DNA elements. Dev Cell.

[83] Ke Y, Xu Y, Chen X, Feng S, Liu Z, Sun Y (2017). 3D chromatin structures of mature gametes and structural reprogramming during mammalian embryogenesis. Cell.

[84] Chen X, Ke Y, Wu K, Zhao H, Sun Y, Gao L (2019). Key role for CTCF in establishing chromatin structure in human embryos. Nature.

[85] Nozawa R, Gilbert N (2019). RNA: nuclear glue for folding the genome. Trends Cell Biol.

[86] Concha II, Urzua U, Yañez A, Schroeder R, Pessot C, Burzio LO (1993). U1 and U2 snRNA are localized in the sperm nucleus. Exp Cell Res.

[87] Sendler E, Johnson GD, Mao S, Goodrich RJ, Diamond MP, Hauser R (2013). Stability, delivery and functions of human sperm RNAs at fertilization. Nucleic Acids Res.

[88] Mondal T, Rasmussen M, Pandey GK, Isaksson A, Kanduri C (2010). Characterization of the RNA content of chromatin. Genome Res.

[89] Kianmehr L, Khazali H, Rajabi-Maham H, Sharifi-Zarchi A, Cuzin F, Rassoulzadegan M (2019). Genome-wide distribution of nascent transcripts in sperm DNA, products of a late wave of general transcription. Cells.

[90] Ben Maamar M, Sadler-Riggleman I, Beck D, McBirney M, Nilsson E, Klukovich R (2018). Alterations in sperm DNA methylation, non-coding RNA expression, and histone retention mediate vinclozolin-induced epigenetic transgenerational inheritance of disease. Environ Epigenetics.

[91] Ben Maamar M, Beck D, Nilsson E, McCarrey JR, Skinner MK (2020). Developmental origins of transgenerational sperm histone retention following ancestral exposures. Dev Biol.

[92] Nilsson EE, Sadler-Riggleman I, Skinner MK (2018). Environmentally induced epigenetic transgenerational inheritance of disease. Environ Epigenetics.

[93] Skinner MK (2014). Endocrine disruptor induction of epigenetic transgenerational inheritance of disease. Mol Cell Endocrinol..

[94] Gou LT, Lim DH, Ma W, Aubol BE, Hao Y, Wang X (2020). Initiation of parental genome reprogramming in fertilized oocyte by splicing kinase SRPK1-catalyzed protamine phosphorylation. Cell.

[95] Skinner MK, Guerrero-Bosagna C (2014). Role of CpG deserts in the epigenetic transgenerational inheritance of differential DNA methylation regions. BMC Genomics.

[96] Nilsson EE, Thorson JLM, Ben Maamar M, Beck D, Skinner MK (2020). Epigenome-wide association study (EWAS) for potential transgenerational disease epigenetic biomarkers in sperm following ancestral exposure to the pesticide methoxychlor. Environ Epigenetics.

[97] Ben Maamar M, Nilsson E, Thorson JLM, Beck D, Skinner MK (2021). Transgenerational disease specific epigenetic sperm biomarkers after ancestral exposure to dioxin. Environ Res.

[98] McBirney M, King SE, Pappalardo M, Houser E, Unkefer M, Nilsson E (2017). Atrazine induced epigenetic transgenerational inheritance of disease, lean phenotype and sperm epimutation pathology biomarkers. PLoS ONE.

[99] Ben Maamar M, Sadler-Riggleman I, Beck D, Skinner MK (2018). Epigenetic transgenerational inheritance of altered sperm histone retention sites. Sci Rep.

[100] Thorson JLM, Beck D, Ben MM, Nilsson EE, McBirney M, Skinner MK (2020). Epigenome-wide association study for atrazine induced transgenerational DNA methylation and histone retention sperm epigenetic biomarkers for disease. PLoS One.

[101] Beck D, Ben Maamar M, Skinner MK (2021). Integration of sperm ncRNA-directed DNA methylation and DNA methylation-directed histone retention in epigenetic transgenerational inheritance. Epigenet Chromatin.

[102] Hoghoughi N, Barral S, Curtet S, Chuffart F, Charbonnier G, Puthier D (2020). RNA-guided genomic localization of H2A.L.2 histone variant. Cells.

[103] Zhang X, Gao F, Fu J, Zhang P, Wang Y, Zeng X (2017). Systematic identification and characterization of long non-coding RNAs in mouse mature sperm. PLoS One..

[104] Lismer A, Siklenka K, Lafleur C, Dumeaux V, Kimmins S (2020). Sperm histone H3 lysine 4 trimethylation is altered in a genetic mouse model of transgenerational epigenetic inheritance. Nucleic Acids Res.

[105] Slyvka Y, Zhang Y, Nowak FV (2015). Epigenetic effects of paternal diet on offspring: emphasis on obesity. Endocrine.

[106] Öst A, Lempradl A, Casas E, Weigert M, Tiko T, Deniz M (2014). Paternal diet defines offspring chromatin state and intergenerational obesity. Cell.

[107] Ornellas F, Carapeto PV, Mandarim-de-Lacerda CA, Aguila MB (2017). Pais obesos levam a metabolismo alterado e obesidade em seus filhos na idade adulta: revisão de estudos experimentais e humanos. J Pediatr (Rio J).

[108] Terashima M, Barbour S, Ren J, Yu W, Han Y, Muegge K (2015). Effect of high fat diet on paternal sperm histone distribution and male offspring liver gene expression. Epigenetics.

[109] Nätt D, Kugelberg U, Casas E, Nedstrand E, Zalavary S, Henriksson P (2019). Human sperm displays rapid responses to diet. PLoS Biol.

[110] Grandjean V, Fourré S, De Abreu DAF, Derieppe MA, Remy JJ, Rassoulzadegan M (2015). RNA-mediated paternal heredity of diet-induced obesity and metabolic disorders. Sci Rep.

[111] Chen Q, Yan M, Cao Z, Li X, Zhang YY, Shi J (2016). Sperm tsRNAs contribute to intergenerational inheritance of an acquired metabolic disorder. Science..

[112] Morgan HL, Paganopoulou P, Akhtar S, Urquhart N, Philomin R, Dickinson Y (2020). Paternal diet impairs F1 and F2 offspring vascular function through sperm and seminal plasma specific mechanisms in mice. J Physiol.

[113] Lesch BJ, Tothova Z, Morgan EA, Liao Z, Bronson RT, Ebert BL (2019). Intergenerational epigenetic inheritance of cancer susceptibility in mammals. Elife.

[114] Subhash S, Kanduri M, Kanduri C (2020). Sperm originated chromatin imprints and LincRNAs in organismal development and cancer. iScience.

[115] Western PS (2018). Epigenomic drugs and the germline: collateral damage in the home of heritability?. Mol Cell Endocrinol..

[116] Rodgers AB, Bale TL (2015). Germ cells origins of Posttraumatic Stress Disorder Risk: the transgenerational impact of parental stress experience. Biol Psychiatry.

[117] Wu L, Lu Y, Jiao Y, Liu B, Li S, Li Y (2016). Paternal psychological stress reprograms hepatic gluconeogenesis in offspring. Cell Metab.

[118] Lei J, Nie Q, Chen DB (2018). A single-cell epigenetic model for paternal psychological stress-induced transgenerational reprogramming in offspring. Biol Reprod.

[119] Gapp K, Jawaid A, Sarkies P, Bohacek J, Pelczar P, Prados J (2014). Implication of sperm RNAs in transgenerational inheritance of the effects of early trauma in mice. Nat Neurosci.

[120] Benito E, Kerimoglu C, Ramachandran B, Pena-Centeno T, Jain G, Stilling RM (2018). RNA-dependent intergenerational inheritance of enhanced synaptic plasticity after environmental enrichment. Cell Rep.

[121] Sandin S, Schendel D, Magnusson P, Hultman C, Surén P, Susser E (2016). Autism risk associated with parental age and with increasing difference in age between the parents. Mol Psychiatry.

[122] Yoshizaki K, Kimura R, Kobayashi H, Oki S, Kikkawa T, Mai L (2021). Paternal age affects offspring via an epigenetic mechanism involving REST/NRSF. EMBO Rep.

[123] Yuan S, Schuster A, Tang C, Yu T, Ortogero N, Bao J (2016). Sperm-borne miRNAs and endo-siRNAs are important for fertilization and preimplantation embryonic development. Dev.

[124] Chen W, Peng Y, Ma X, Kong S, Tan S, Wei Y (2020). Integrated multi-omics reveal epigenomic disturbance of assisted reproductive technologies in human offspring. EBioMedicine.

[125] Štiavnická M, García-Álvarez O, Ulčová-Gallová Z, Sutovsky P, Abril-Parreño L, Dolejšová M (2020). H3K4me2 accompanies chromatin immaturity in human spermatozoa: an epigenetic marker for sperm quality assessment. Syst Biol Reprod Med.

[126] Zhang Z, Mu S, Chen T, Sun Z, Shu Z, Li Y (2020). H4S1ph, an alternative epigenetic marker for sperm maturity. Andrologia.

[127] Xu H, Wang X, Wang Z, Li J, Xu Z, Miao M (2020). MicroRNA expression profile analysis in sperm reveals hsa-mir-191 as an auspicious omen of in vitro fertilization. BMC Genomics.

[128] Cheung S, Parrella A, Rosenwaks Z, Palermo GD (2019). Genetic and epigenetic profiling of the infertile male. PLoS One.

[129] Pértille F, Alvarez-Rodriguez M, da Silva AN, Barranco I, Roca J, Guerrero-Bosagna C (2021). Sperm methylome profiling can discern fertility levels in the porcine biomedical model. Int J Mol Sci.

[130] Nixon B, De Iuliis GN, Dun MD, Zhou W, Trigg NA, Eamens AL (2019). Profiling of epididymal small non-protein-coding RNAs. Andrology.

[131] alas-Huetos S, Blanco A, Vidal J, Grossmann F, Pons M, Garrido MC (2016). Spermatozoa from normozoospermic fertile and infertile individuals convey a distinct miRNA cargo. Andrology.

[132] Jodar M, Selvaraju S, Sendler E, Diamond MP, Krawetz SA (2013). The presence, role and clinical use of spermatozoal RNAs. Hum Reprod Update.

[133] Mashoodh R, Habrylo IB, Gudsnuk KM, Pelle G, Champagne FA (2018). Maternal modulation of paternal effects on offspring development. Proc R Soc B Biol Sci.

[134] Torres-Flores U, Hernández-Hernández A (2020). The interplay between replacement and retention of histones in the sperm genome. Front Genet.

